# Editorial: Machine learning and big data analytics in mood disorders

**DOI:** 10.3389/fpsyt.2024.1406826

**Published:** 2024-04-17

**Authors:** Lu Yang, Jun Chen

**Affiliations:** ^1^ Department of Community Health and Epidemiology, Faculty of Medicine, Dalhousie University, Halifax, NS, Canada; ^2^ Clinical Research Center, Shanghai Mental Health Center, Shanghai Jiao Tong University School of Medicine, Shanghai, China; ^3^ Shanghai Key Laboratory of Psychotic Disorders, Shanghai, China

**Keywords:** machine learning (ML), big data & analytics, mood disorders, pharmacological treatment, biological heterogeneity, post-stroke depression, prescriptions pattern, pain-related affective and cognitive behavior

Advances in information technology and devices enabled generating, processing, and storage of unimaginable amounts of data, which launched the big data era (1). Over the last decades, big data has firmly established itself in psychiatric research and practice (2). Mood disorders are a group of psychiatric conditions that are marked by conspicuous disturbances in emotional disposition (i.e., extreme lows called depression or highs called mania/hypomania), leading to significant mental and physical disturbances and substantial contribution to the global disease burden.

However, the diagnosis of mood disorders solely relied on examination of subjective symptoms, with no established biomarker that plays a definitive role in the pathogenesis (3). There are persistent arguments that traditional psychiatric taxonomy just identifies a group of individuals with biological heterogeneity underlying various phenotypes (4). With the evolution of machine learning (ML) techniques and increasing sources of real-world and bioinformatics data, psychiatrists and investigators now have an unprecedented opportunity to understand psychiatric disorders better and improve clinical practice (5). ML and big data analytics have great potential to advance psychiatric research from refining taxonomy, personalized therapy, and intelligent drug design to population screening and electronic health record (EHR) mining (6, 7).

There is an increasing number of studies which applied ML and big data analytics in the detection, diagnosis, and treatment of mental disorders. To advance research in this area effectively, we organized a Research Topic aimed at presenting the forefront advances and potential scenarios of application ML and big data analytics in research on mood disorders.

In the paper entitled “*Investigation of the pharmacological treatment patterns of Chinese patients with major depressive disorder under real-world settings using multi-channel sequence analysis*”, Wu et al. present a study describing the longitudinal pharmacological treatment patterns of major depressive disorder (MDD) patients using EHR from a major psychiatric hospital in China. This study provided valuable information on the real-world pharmacological treatment practice of MDD, considering chronological sequences, cumulative treatment steps, and polypharmacy usage, contributing to the insights in bridging the guideline-practice gap in the pharmacological treatment of MDD.

The paper “*Dissecting clinical and biological heterogeneity in clinical states of bipolar disorder: a 10-year retrospective study from China*” by Zhu et al. presented a retrospective study which investigated the biological heterogeneity in clinical states of bipolar disorder (BD) using EHR data from one of a largest medical center of West China. By using explainable ML techniques, this study identified and mapped neuropsychological symptomatology, comorbidity, vital signs, and blood laboratory indicators that can predict distinct BD states. These findings contributed to a better understanding of the clinical, physiological, and biological heterogeneity among BD clinical states.

Another paper from the Chinese group by Chen et al. entitled “*Predicting new-onset post-stroke depression (PSD) from real-world data using machine learning algorithm*” developed ML models to predict new-onset post-stroke depression from real-world data. This study identified important factors to alert clinicians for early detection of depression in high-risk stroke patients. It supported that ML models can provide potential predictive tools for PSD.

Takano et al.‘s paper, entitled “*Clinical characteristics and prescriptions associated with a 2-year course of rapid cycling and euthymia in bipolar disorder: a multicenter treatment survey for bipolar disorder in psychiatric clinics*” investigated the influence of patient backgrounds and prescription patterns on the progression of rapid cycling (RC) and euthymia (EUT) using a large sample from the first and second iterations of a multicenter treatment survey for BD in psychiatric clinics (MUSUBI). This study supported and extended previous findings on the distinctive risk factors of RC and EUT of BD concerning the prognosis.

The paper entitled “*Identifying circulating biomarkers for major depressive disorder*” by Zhang et al. explored the circulating biomarkers for MDD diagnosis from 440 circulating cytokines by LASSO regression. The diagnostic value of the identified six MDD-related characteristic proteins in this study showed a moderate performance in the discrimination between MDD and health control. These results supported the diagnostic value of circulating proteins, including insulin, CD40L, CD155, Lipocalin-2, HGF, and LIGHT for MDD patients.

In the paper entitled “*Research hotspots and trends on neuropathic pain-related mood disorders: a bibliometric analysis from 2003 to 2023*”, Wang et al. analyzed the hotspots and trends in neuropathic pain (NP)-related mood disorder research using bibliometric methods. This study provided insight into the current state and trends in NP-related mood disorder research over the past 20 years. This study enabled researchers to identify new perspectives on potential collaborators and cooperative institutions, hot topics, and research frontiers in this field.

Taken together, the papers of this Research Topic are, in our opinion, timely and innovative, underlining how ML and big data analytics might advance the research on mood disorders. We recommend collaborative teamwork involving professionals in multidisciplinary fields, including but not limited to clinical psychiatry, information technology, and biological statistics, to foster scientific progress in understanding and management of mood disorders. It is also essential to combine data-driven and theory-driven perspectives in the era of psychiatric research (8).

## Author contributions

LY: Investigation, Validation, Writing – original draft. JC: Conceptualization, Funding acquisition, Investigation, Methodology, Project administration, Resources, Supervision, Writing – review & editing.

## References

[1] Kudyba S Kwatinetz M. Introduction to the big data era. Big data mining analytics. (2014), 1–17. doi: 10.1201/b16666

[2] Dwyer DB Falkai P Koutsouleris N. Machine learning approaches for clinical psychology and psychiatry. Annu Rev Clin Psychol. (2018) 14:91–118. doi: 10.1146/annurev-clinpsy-032816-045037 29401044

[3] Kennis M Gerritsen L van Dalen M Williams A Cuijpers P Bockting C. Prospective biomarkers of major depressive disorder: a systematic review and meta-analysis. Mol Psychiatry. (2020) 25:321–38. doi: 10.1038/s41380-019-0585-z PMC6974432 31745238

[4] Ratheesh A Berk M Schmaal L. Can we overcome the heterogeneity of mood disorders in clinical trials? London, England: SAGE Publications Sage UK (2023) p. 309–11. 10.1177/00048674231158266 36786223

[5] Bzdok D Meyer-Lindenberg A. Machine learning for precision psychiatry: opportunities and challenges. Biol Psychiatry: Cogn Neurosci Neuroimaging. (2018) 3:223–30. doi: 10.1016/j.bpsc.2017.11.007 29486863

[6] Dash S Shakyawar SK Sharma M Kaushik S. Big data in healthcare: management, analysis and future prospects. J big Data. (2019) 6:1–25. doi: 10.1186/s40537-019-0217-0

[7] Cearns M Hahn T Baune BT. Recommendations and future directions for supervised machine learning in psychiatry. Trans Psychiatry. (2019) 9:271. doi: 10.1038/s41398-019-0607-2 PMC6805872 31641106

[8] Rutledge RB Chekroud AM Huys QJ. Machine learning and big data in psychiatry: toward clinical applications. Curr Opin Neurobiol. (2019) 55:152–9. doi: 10.1016/j.conb.2019.02.006 30999271

